# Concussion in Women's Flat-Track Roller Derby

**DOI:** 10.3389/fneur.2022.809939

**Published:** 2022-02-14

**Authors:** Melissa D. Stockbridge, Zafer Keser, Rochelle S. Newman

**Affiliations:** ^1^Department of Hearing and Speech Sciences, University of Maryland, College Park, MD, United States; ^2^Department of Neurology, Johns Hopkins University School of Medicine, Baltimore, MD, United States; ^3^Department of Neurology, Mayo Clinic, Rochester, MN, United States

**Keywords:** concussion, cognition, language, roller derby, psychometric assessment

## Abstract

Concussions are common among flat-track roller derby players, a unique and under-studied sport, but little has been done to assess how common they are or what players can do to manage injury risk. The purpose of this study is to provide an epidemiological investigation of concussion incidence and experience in a large international sampling of roller derby players. Six hundred sixty-five roller derby players from 25 countries responded to a comprehensive online survey about injury and sport participation. Participants also responded to a battery of psychometric assessment tools targeting risk-factors for poor injury recovery (negative bias, social support, mental toughness) and players' thoughts and feelings in response to injury. Per 1,000 athletes, 790.98 concussions were reported. Current players reported an average of 2.2 concussions, while former players reported 3.1 concussions. However, groups were matched when these figures were corrected for differences in years of play (approximately one concussion every 2 years). Other frequent injuries included fractures in extremities and upper limbs, torn knee ligaments, and sprained ankles. We found no evidence that players' position, full-contact scrimmages, or flooring impacted number of concussions. However, neurological history and uncorrected vision were more influential predictors of an individual's number of concussions during roller derby than years of participation or age, though all four contributed significantly. These findings should assist athletes in making informed decisions about participation in roller derby, though more work is needed to understand the nature of risk.

## Introduction

Concussion is defined as “a pathophysiological disturbance in neurologic function characterized by clinical symptoms induced by biomechanical forces” per American Academy of Neurology (1) and up to one-third result in long-term effects (2). Most research on sports-related concussions has focused on a small subset of sports, often utilizing professional athletes (e.g., the National Football League (3); or college-aged athletes, most of whom tend to be men^1^.

Recent studies have examined sex and gender differences in symptomatology and recovery after concussion. Female athletes with comparable injuries report increased post-concussion symptoms both within and beyond 1 week and longer recovery times (4, 5). There is some debate as to whether differences are due to biological sex, gender, or other factors (e.g., access to care) (6) that may covary or co-occur (7). The focus of the current descriptive epidemiological study is roller derby, a sport most often played non-professionally by women beyond college-age who are often remarkably diverse in body type, sexuality and gender identity, and socioeconomic status (8, 9). Because of the decentralized culture of roller derby, it is difficult to make clear estimates of the number and demographic characteristics of active players. The Women's Flat Track Derby Association (WFTDA), the largest of seven main international governing bodies for the sport of flat track roller derby, presently boasts 447 member leagues on six continents, but an estimated 758 leagues exist worldwide amidst the pandemic in 2021 (http://flattrackstats.com/). Each league in WFTDA is required to have at least 15 skaters, but many leagues have multiple teams, each of which have 10–20 skaters. These factors make roller derby dissimilar from the sports played by women that most frequently are studied in the literature on concussions. However, concussion is one of the most common injuries associated with roller derby (10), along with sprains and torn knee ligaments.

To make informed decisions about their participation in sport and assumption of risk, athletes need to know how common injuries are and what choices they can make that may modify their risk of injury. Prior studies have highlighted the potential for a high incidence of concussion in roller derby, despite remarkably limited work directly addressing the sport from a medical perspective. Lane et al. (11) retrospectively examined 1 year of discharged patients with closed head injury or concussion from the University of Arizona Emergency Department and found that 6.5% of all sports-related concussions (*N* = 279) were the result of “roller derby, roller skating, and skateboarding.” This was greater than sports-related concussion from soccer (5%). It was only exceeded by cycling (30%), football (12%), basketball (12%), and horseback riding (8%), sports studied considerably more often within concussion literature.

The two prior investigations into concussion incidence in roller derby had considerable limitations. Cathorall and Peachey (12) found that 4% of 1,395 players had a concussion in the past year, but the authors did not consider participation across multiple years. Hoskins & Hooker (13) compiled 74 responses to a tablet-based survey distributed at the 2014 RollerCon roller derby convention in Las Vegas. They found that 54.1% of athletes had experienced one or more concussions (1 concussion per player over 3.6 years of participation, with 10% reporting three or more), but noted the need for a larger sample size to better estimate concussion in the sport. The first goal of our work was to reexamine these estimates in a international sample of roller derby players in order to describe injury rates among roller derby athletes more thoroughly.

Minimal information has been published on the modification of injury risk in the sport of roller derby. Typically, we examine both the risk of concussion associated with a given sport generally (14) and the effects of decisions such as rule changes (15–18) and the introduction of safety equipment (19, 20). The WFTDA dictates safety parameters of the play environment and how concussions may be assessed at the sidelines to determine whether an individual may safely continue to participate. They require all skaters, including skating officials, wear wrist guards, elbow and knee pads, mouth guards, and helmets with a hard-protective shell designed for skating (21). Pauelsen (10), in an undergraduate thesis, surveyed 540 players predominantly in Sweden and other countries in Europe and identified multiple risk factors for increased rates of injury in roller derby, including the number of years played and the number of bouts, or matches, per year, contact scrimmages during training, flooring type, and position, with the highest risk associated with the role of the jammer. The WFTDA recommends playing on sport court or concrete (21). Differences in safety also have been noted among body types and levels of fitness (22). Individual differences in health status and history previously have not been examined as potential contributors to increased risk of injury in roller derby. The second goal of our work was to replicate and elaborate on the findings from Pauelsen to include both sport-related factors and personal factors in order to better understand the nature of injury risk in roller derby.

Finally, given the rich literature examining roller derby psychology and culture (23–31), we used this opportunity to take a first look within the context of roller derby at features of personality, temperament, and affect that are known to carry enhanced risk for poor recovery from injury. Dispositional negativity, the propensity for more frequent, intense, and enduring negative affect (32–35), is a risk factor for poor brain injury recovery (36–38). Individuals with high negativity both *experience* and *report* a magnified reactivity to negative events or other stressors, further elevating their overall level of disability and perception of disability (39). In contrast, other personal factors have demonstrated a protective effect against stressor reactivity and poor response to trauma. For example, perceived social support is associated with reduced risk associated with dispositional negativity (40, 41). Mental toughness also has garnered attention as a protective factor against poor recovery (42), including in roller derby players (43), where authors have noted a complex relationship between toughness, positive injury psychological response, and physical injury exacerbation.

Taken together, the broader purpose of this research was to examine a large international sampling of roller derby players in order to provide a thorough and updated look into concussion incidence and experience by players of roller derby, a high-intensity, under-studied sport.

## Materials and Methods

### Recruitment

All work was conducted with the formal approval of the University of Maryland Institutional Review Board. Data were collected remotely through Qualtrics experience management software tools distributed through social media across online roller derby communities. Methods of data collection were designed to mitigate in-person barriers to sampling, such as transportation and lengthy in-person interviews. Remote behavioral data collection methods, similar to those proposed here, are increasingly common for studies of concussion and other medical conditions and produce comparable results to in-person testing (44–48).

The survey received 1,489 clicks, between February 9, 2018 and November 30, 2019, from 25 countries, including 47 states of the United States. This captured 665 roller derby players who provided the minimum information necessary to be included in analysis (i.e., roller derby participation and concussion endorsement; Table 1).

**Table 1:** Sample description and demographics by roller derby participation.

	**Current (*N* = 535)**	**Past (*N* = 130)**	**Test statistic**
Age (years)	32.66 ± 7.26	35.48 ± 7.20	t (663) = 3.99, *p* < 0.001
Gender (see text footnote 1)			χ^2^ = 1.87, *p* = 0.802
Women	481	120	
Men	25	3	
Non-binary/Other	29	7	
Trans*	4	2	
Education (years)	16.11 ± 1.97	15.97 ± 1.85	t (632) = 0.76, *p* = 0.449
Years of roller derby	4.56 ± 2.65	5.81 ± 4.60	t (150) = 2.97, *p* = 0.004^†^
Practices per month	10.12 ± 6.42	10.02 ± 4.27	t (660) = 0.16, *p* = 0.872
Bouts per year	9.30 ± 8.59	12.23 ± 27.40	t (133) = 1.19, *p* = 0.235^†^
Concussion endorsement			
Recent	31(39)	3	χ^2^ = 13.33, *p* = 0.002
History	384(72)	108(8)	χ^2^ = 7.64, *p* = 0.023
None	79	14	
Concussions per player	2.16 ± 1.95	3.08 ± 2.46	t (155) = 3.72, *p* < 0.001^†^
Concussions per player per year	0.63 ± 0.79	0.67 ± 0.68	t (573) = 0.45, *p* = 0.656
Significant hits per player	16.90 ± 27.02	25.24 ± 34.14	t (112) = 1.51, *p* = 0.134^†^
Somatic symptoms	20.00 ± 15.94	25.83 ± 18.73	t (162) = 3.16, *p* = 0.002^†^
BRS (/5)	3.43 ± 0.77	3.09 ± 0.84	t (455) = 3.66, *p* < 0.001
BFI-2 Ne (/100)	50.32 ± 10.09	52.41 ± 10.94	t (440) = 1.69, *p* = 0.091
MSPSS (/7)	5.63 ± 1.07	5.41 ± 1.16	t (85) = 1.32, *p* = 0.192^†^
SMTQ (/42)	38.22 ± 4.37	38.65 ± 3.96	t (85) = 1.14, *p* = 0.259^†^
IEQ (/48)	21.21 ± 8.74	25.00 ± 22.63	t (1) = 0.24, *p* = 0.852^†^
IPQ-R (/5)	2.99 ± 0.45	3.12 ± 0.62	t (101) = 1.73, *p* = 0.087^†^

*All statistics are two-tailed*.

† Degrees of freedom corrected, as equal variances assumption violated. Data are presented as counts or mean ± standard deviation. Suspected concussions are included in parentheses. Recency was defined as within 30 days. Trans* individuals were included in statistics of the gender identity that they identified. BRS, Brief Resilience Scale; BFI-2 Ne, Big Five Inventory 2 negative emotionality t-score; MSPSS, Multidimensional Scale of Perceived Social Support; SMTQ, Sports Mental Toughness Questionnaire; IEQ, Injustice Experience Questionnaire; IPQ-R, adaptation of the Revised Illness Perception Questionnaire referencing concussion.

### Data Collection

Surveys utilized a mixture of closed- (i.e., yes/no, ratings) and open-ended responses. Respondents were asked about their participation in roller derby and other sports with a closed set of responses (“Yes, I currently participate.”; “I have participated previously, but don't currently.”; “I have never participated.”). They also were asked how many years they participated, what role or positions they typically played, how many bouts they participated in per year, and how many practices they participated in per month. They were asked how often scrimmages were full contact and about flooring in the arena where they practiced most frequently. In addition to an open-ended prompt to list all other sports athletes participated in, we also asked if respondents participated in any other activities that may involve head impacts or other rough physical activity, and if so, for how many years.

In addition to age and gender identity, participants were asked numerous questions about their medical history and recent medical events. Respondents were given a standard definition of concussion and asked to report if they had had one while playing roller derby in the past 30 days or at all and how it was determined that they did. Individuals who only suspected they had a concussion, but it was not verified sideline or by a medical professional, were noted. Since neither the athlete nor an outside medical professional was able to verify a suspected concussion, these were excluded from analysis (though this makes it possible that true concussions would thus be underestimated). Athletes were asked to describe the incident or incidents, part of head that was hit, and whether they were unconscious and for how long (if known). Athletes provided rich medical histories of both past head injuries and other significant injuries and rated their somatic symptoms using a widely available tool (49). Significant hits and concussions were artificially capped at 100, despite some participants endorsing 500 or more hits they considered significant in their lifetime (impacting *N* = 22 [3%] participants whose estimates were >100). The concept of “significance” in this context was left deliberately subjective, though narratives suggested “significance” generally aligned with requiring medical attention resulting in identifiable injury. They also were asked questions about lifetime learning disability, ADD/ADHD, speech language disorder, fluency disorder, dyslexia, epilepsy, their current medications, and their family history of cognitive or language diagnoses. Finally, we asked questions about menstruation, menopause, and pregnancy.

Respondents were asked to complete a number of psychometric assessments selected to capture aspects of their thoughts and feelings in general and relative to roller derby: the Big Five Inventory 2 negative emotionality items [BFI 2-Ne; (50)], Multidimensional Scale of Perceived Social Support [MSPSS; (51)], Brief Resilience Scale [BRS; (52)], Sports Mental Toughness Questionnaire [SMTQ; (53)], Injustice Experience Questionnaire [IEQ; (54)], and an adaptation of the Revised Illness Perception Questionnaire [IPQ-R; (55)], referencing concussion. These tools target sources of risk for poor injury recovery. The BFI-2 examines differences in personality and temperament by having respondents rate their level of agreement with 60 statements about themselves (e.g., “I am someone who… worries a lot”) on a 5-point scale. T-scores were calculated from the mean rating on the 12 items that make up the Ne subscore (50), with higher values indicating greater negative emotionality. The MSPSS examines the strength of supportive relationships, another potential risk factor for poor response to trauma. Respondents rate their level of agreement with 12 statements on a 7-point scale and a mean score is used for interpretation (1-2.9: low support, 3-5: moderate, 5.1-7: high). The BRS consists of 6 statements respondents rate for agreement on a 5-point scale. Higher mean rating is associated with greater resilience. The SMTQ examines mental toughness in three factors: confidence, constancy, and control using 14 statements, each rated on a 0–3 scale. Ratings are summed for interpretation (maximum score = 42), with higher toughness associated with higher scores. The IEQ captures how frequently an individual has feelings of severe and irreparable injury-related loss, unfairness, and external attribution of blame, following an injury (54). Respondents rate how frequently they experience 12 thoughts or feelings when thinking about injuries from “Never” 0 to “All the time” 4 (maximum score = 48), with higher scores associated with a more negative outlook. The 35-item IPQ-R captures participants' responses to injury across time, consequences, treatment, and feelings of control through their ratings of agreement with statements about these facets of experience on a 5-point scale, with higher scores associated with poorer adjustment.

Finally, athletes completed an extensive battery of cognitive and linguistic tasks designed to capture areas of weakness following concussion. These data were described elsewhere (56) and raw data are publicly available in the TBIBank (57).

### Statistical Analysis

All statistical analyses were conducted in IBM SPSS Statistics version 28. Reported concussions and orthopedic injuries were counted and calculated out of a standard 1,000 players. Current and past players were examined separately, as it was of interest to consider what conditions were associated with departure from the sport. Demographic variables, participation characteristics, concussion history, and psychometric assessments were examined using t-tests, except in cases where assumptions of normality were violated, in which case corrected calculations are noted. Correlations (Pearson's r) are reported as they further describe associations among key facets of interest, though these are provided for exploratory purposes only.

In order to examine risk of concussion, we considered three previously-examined salient features of roller derby participation: the position of the player, frequency of full-contact scrimmages, and kind of flooring played on most frequently (10). While it was initially planned that each of these would be examined using analyses of variance, position was instead analyzed using independent t-tests, as many athletes stated they played multiple positions (thus, the categories were not mutually exclusive). Next, important differences in demographics (age and years of play) as well as self-reported medical history: childhood developmental diagnoses (learning disability, ADD/ADHD, speech-language disorder, fluency disorder, dyslexia), significant neurological history (i.e., reported incidents of neurological conditions more severe than concussion, such as moderate-serious TBI and epilepsy), and visual disturbance were entered into regression analyses to arrive at two models.

## Results

### Sample Description

**Figure 1:**
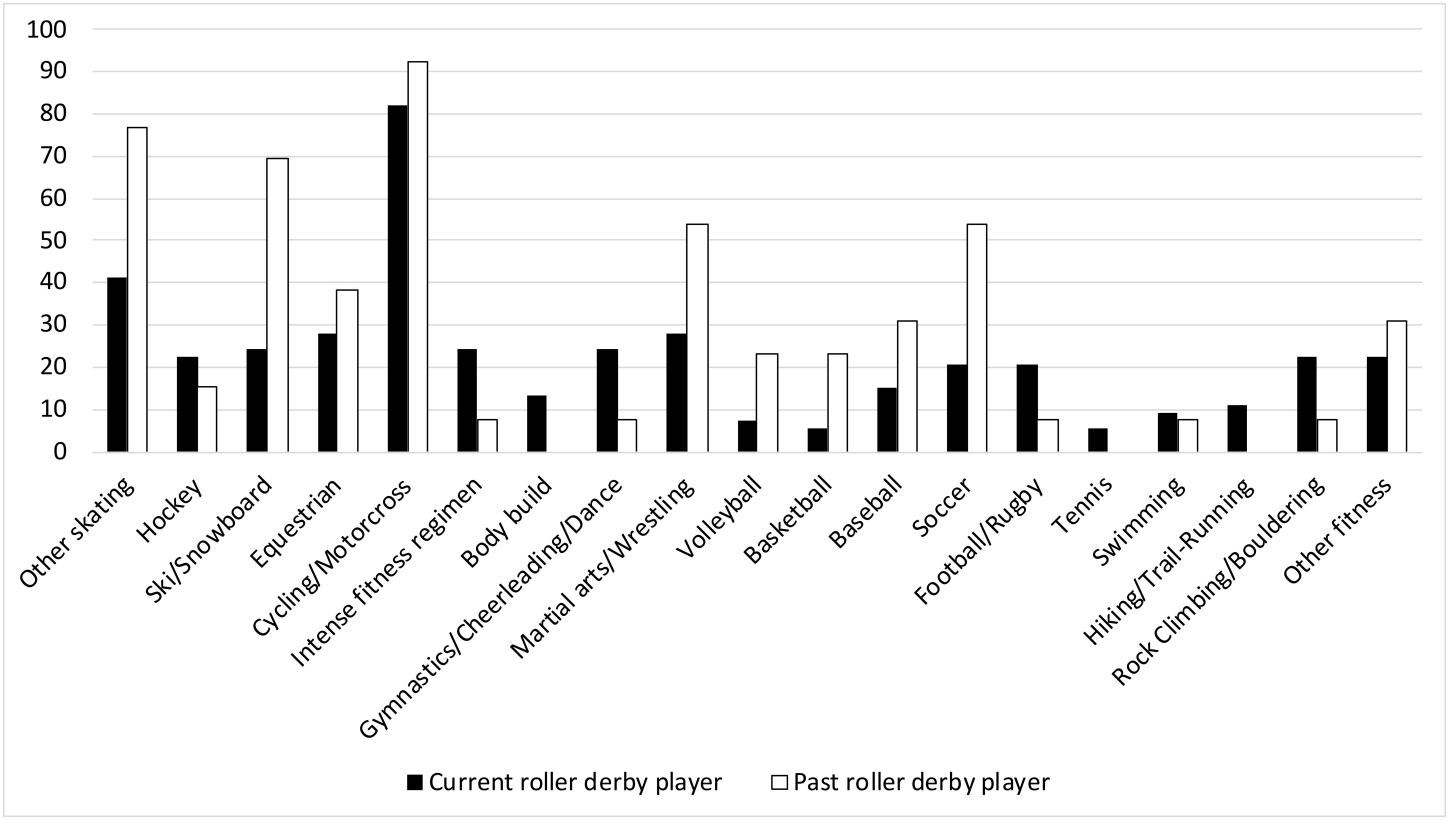
Sport participation outside of roller derby per 1,000 athletes. Other skating: skatepark users, bowl skating, and ice skating. Intense fitness regimens: Tough Mudder, Spartan Races, CrossFit, etc. Other fitness: golf, field hockey, lacrosse, surfing, Quidditch, contact Live Action Role Play (LARPing), kayak polo, ultimate frisbee, paintball, etc.

The 665 roller derby players who responded to the survey are described in Table 1. Former roller derby players were older than current players (t (663) = 3.99, *p* < 0.001) and had played significantly longer, by approximately 1 year (Levene's F = 4.10, *p* = 0.04; t (149.11) = 2.97, p=0.004). Players engaged in a similar number of practices per month (t (660) = 0.16, *p* = 0.872) and bouts per year (Levene's F = 10.55 =0.001; t (133.04) = 1.19, *p* = 0.235). Two-thirds of athletes played a single position, while the remainder switched between roles (block: 69.92%, jam: 37.41%, pivot: 24.24%). Over 80% of athletes said that all scrimmages were full contact. Many players noted anecdotally that injuries occurred during practice, not bouts. The majority of play was on “sport court,” an engineered wood flooring product recommended by the WFTDA, or wood (57.7%), while just over a third played most frequently on concrete. Derby players frequently also participated in a variety of other sports; 139 current players (26%) and 43 past players (33%) played at least one additional sport (Figure 1). Past players reported significantly lower resilience than current players of roller derby (BRS; t (455) = 3.66, *p* < 0.001). They expressed similar levels of dispositional negativity (BFI 2-Ne), social support (MSPSS), mental toughness (SMTQ), perceived injustice following injury (IEQ), and perception of injury (IPQ-R).

**Figure 2:**
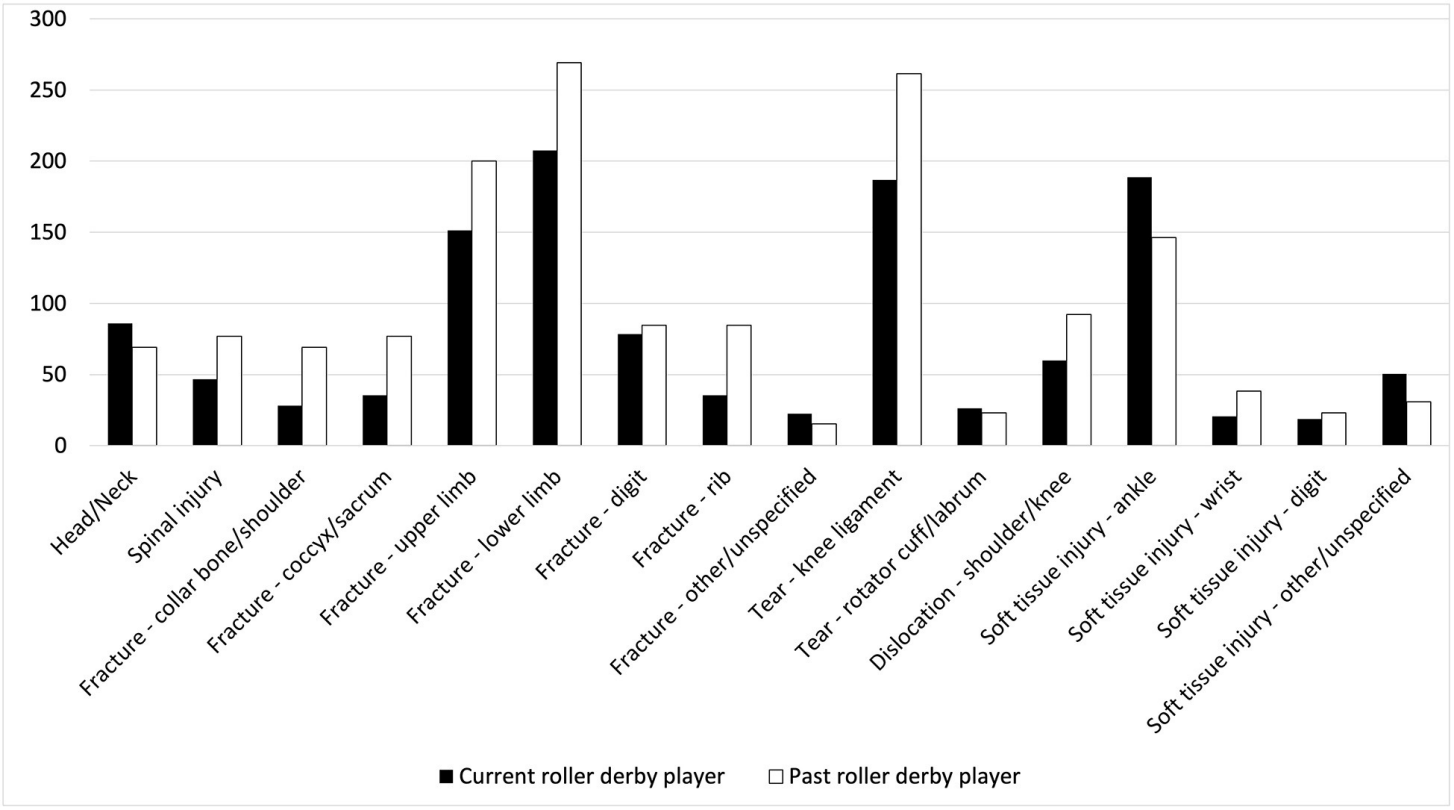
Orthopedic injury history endorsement during roller derby per 1,000 athletes. Head/Neck: whiplash, skull fracture, broken nose; digit: fingers, toes; upper limb: arm, elbow, wrist, hand; lower limb: hip, pelvis, leg, ankle, foot; Soft tissue injury: tears, sprains, dislocation.

**Figure 3:**
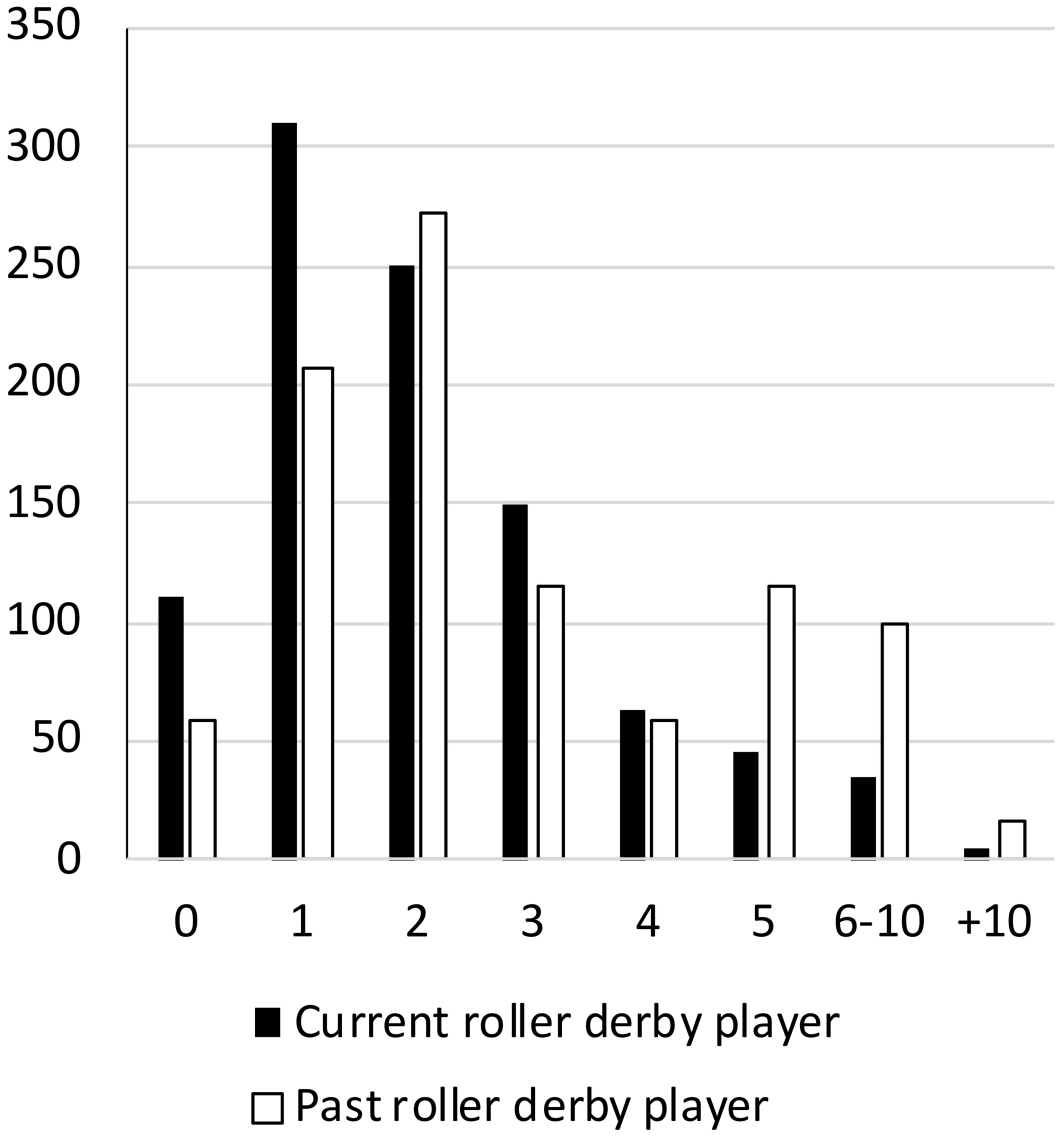
Number of concussions per player during roller derby per 1,000 athletes.

Per 1000 athletes, 775.70 concussions were reported among current and 853.85 among former roller derby players (790.98 overall), not accounting for suspected concussions. Only 9/34 recent concussions were that individual's first concussion; the majority had at least one prior concussion. Current and former players described similar numbers of significant hits (Levene's F = 33.69, *p* < 0.001; t (112.15) = 1.51, *p* = 0.13). All athletes endorsed substantial orthopedic injury history (Figure 2). Former players reported more concussions than current players (Figure 3), by approximately one (Levene's F = 14.14, *p* < 0.001; t (154.94) = 3.72, *p* < 0.001, d = 0.41). However, this was no longer significant when adjusted for the number of years individuals had played (concussions/years; t (662) = 0.79, *p* = 0.428). Higher numbers of concussions were associated significantly with greater mental toughness (r = 0.15, *p* = 0.005), and perceptions of injury (r = 0.17, *p* = 0.002) and showed weaker evidence of association with dispositional negativity (r = 0.12, *p* = 0.03) and perceived injustice following injury (r = 0.28, *p* = 0.04).

To explore risk of concussion, we considered three salient features of roller derby participation: the position of the player, frequency of full-contact scrimmages, and kind of flooring played on most frequently using *t*-tests and analysis of variance. None of these were statistically associated with greater numbers of concussions among players (Table 2), which hovered consistently around two concussions.

**Table 2:** Concussions by bout characteristics.

	**Concussions**	**Test statistic**
Position		
Jammer	2.39 ± 2.31	t (574) = 0.360, *p* = 0.710
Blocker	2.4 ± 2.07	t (574) = 0.929, *p* = 0.353
Pivot	2.23 ± 1.55	t (421) = 0.986, *p* = 0.325
Court		F (4,569) = 1.409, *p* = 0.230
Wood	2.52 ± 2.1	
Cement	2.44 ± 2.34	
Sport court	2.15 ± 1.79	
Rubber/Plastic	2.31 ± 1.90	
Other/Unknown	1.69 ± 1.08	
Full-Contact scrimmages		t (569) = 0.471, *p* = 0.638
Always	2.34 ± 2.10	
Sometimes	2.48 ± 2.09	

*All statistics are two-tailed*.

Next, age and years of roller derby participation were entered into a hierarchical multiple regression analysis predicting the total number of concussions among roller derby players (Model 1) as these were demographic features with strong anticipated (and demonstrated) associated with concussions (age; r = 0.123, *p* = 0.003; years: r = 0.113, *p* = 0.006), followed by the addition of clinical comorbidities divided into developmental diagnoses, significant neurological history, and reported uncorrected visual disturbance coded dichotomously (Model 2; Table 3).

**Table 3:** Concussions by demographic and medical history characteristics.

	**Concussions**	**Test statistic**
Developmental disability		t (574) = 2.20, *p* = 0.03, d = 0.84
Endorsed	4.07 ± 2.59	
Not endorsed	2.33 ± 2.08	
Neurological history		t (22) = 2.25, *p* = 0.034, d = 0.68^†^
Endorsed	3.70 ± 2.96	
Not endorsed	2.29 ± 2.03	
Visual disturbance		t (36) = 2.44, *p* = 0.02, d = 0.598^†^
Endorsed	3.51 ± 2.97	
Not endorsed	2.27 ± 2.01	

*All statistics are two-tailed*.

† *Degrees of freedom corrected, as equal variances assumption violated. Data are presented as counts or mean ± standard deviation*.

The first model was significant in predicting total number of concussions (R^2^ = 0.02, F (2, 572) = 6.52, *p* = 0.002), with both age (t = 2.35, *p* = 0.02, β = 0.03) and years of roller derby participation (t = 2.08, p = 0.04, β = 0.06) contributing significantly to the prediction. The second model also was significant (R^2^ = 0.07, F (5, 569) = 7.85, *p* < 0.001) and improved on the previous model (F (3, 569) = 8.57, *p* < 0.001). Age remained a significant predictor (*t* = 2.02, *p* = 0.04, β = 0.02), as did years of participation (t = 1.99, *p* = 0.05, β = 0.05) when using the second model. Among clinical comorbidities, significant neurological history (t = 3.16, =0.002, β = 1.38) and visual disturbance (t = 3.58, *p* < 0.001, β = 1.28) were significant, whereas a history of developmental disability was not (t = 1.89, *p* = 0.06, β = 1.47). These factors were associated with greater influence over total number of concussions than years of play or age.

## Discussion

In this study, we investigated the prevalence of concussion and other injuries in roller derby and examined risk factors for injury in the sport. We also explored the affective risk and protective factors of poor injury recovery among players.

Concussions are quite common in roller derby. By our estimation in the present study, most players get one or two concussions during their time in the sport. Only around 10% of current roller derby players had not had a concussion, and that figure nearly halved by players' departure. These findings answer a slightly different question than the one pursued by Cathorall and Peachey (12), who found that 4% of players had a concussion in the past year. Our estimate of players who had a concussion in the past 30 days was 6% (31/535), suggesting that estimates of concussions within the year would be much higher. Our estimates also were higher than those from Hoskins & Hooker (13). There are a number of potential explanations for this discrepancy. Advertisements for this study specifically referenced concussion in roller derby and may have resulted in an over-estimate of concussion in the sport. However, concussion education within the leagues has expanded greatly each year, as have guidelines for sideline concussion monitoring and care. Thus, earlier estimates may have under-appreciated the rate of concussion in the sport. Fractured fingers, toes, upper limb and ankle bones, torn knee ligaments, and ankle sprains and tears were the most commonly reported orthopedic injuries experienced by around a fifth of current players. This is grossly consistent with the findings reported in Pauelsen (10), who found sprains, torn knee ligaments, and concussions as most frequent.

We also explored how dimensions of personality and temperament associated with differences in the patterns of injury among players. While past players were significantly less resilient, they rated themselves like current players in all other examined dimensions – negative emotionality, social support, toughness, perceived injustice, and self-perception with injury. This is concordant with prior work on the culture of roller derby as a sport that tends to attract individuals that are very diverse in certain ways, but tend to share certain values and other traits (24). The number of concussions was associated with greater endorsement of toughness, injury perception, perceived injustice following injury, and a tendency toward negative outlook. More work is needed to understand these relationships, but they may reflect the same underlying effect described by Madrigal et al. (43). The authors found that injured roller derby players, unlike comparable rugby players, were more likely to feel isolated, restless, and cheated after injury and perceived these injuries more negatively in general, as they presented a threat to continued membership in the “sisterhood” that roller derby represents. Injury presented a significant psychosocial and emotional risk to derby players. The authors suggest this may incentivize roller derby players to rejoin play before they are fully healed, thus contributing to more severe, compounded injuries. Policing this behavior in regards to concussion is more complicated than with orthopedic injury, and strategies athletes may use to return to play and rejoin their community despite concussion (i.e., “sandbagging”) are well documented among athletes in many other high-pressure sports (58, 59).

These data did not support the complex landscape of play-related risk factors identified in Pauelsen (10). While Pauelsen also used retrospective online survey methodology, minimal information is available to permit critical evaluation of the discrepancies (60). It appears the data were predominantly from European players, which may not have generalized to the predominantly American players in the present sample. While number of years in play was associated with an increased number of concussions, the number of bouts, nature of contact, flooring type, and position all yielded no relationship with concussion prevalence in our sample. Circumstances of play tend to lead to around two concussions on average for current and past players, with significant variability noted (combined M = 2.35, SD = 2.09). In contrast, the primary risk factor for increased concussion in our data appears to be the player's own prior medical history, particularly a history of significant neurological events (such as a history of seizures or moderate TBI) or uncorrected visual disruption. Unlike facets of gameplay (flooring, contact during scrimmages, number of bouts per year), which are often linked to participation in a certain league or player position, these facets of medical history are individual in nature. Fortunately, these generally are also known to the player, who can enter these into her own calculus about the acceptability of risk associated with participation in this sport.

Our study is limited as the data collection solely relied on remotely filled surveys from the participants. While some studies using survey methods have followed respondents over multiple sessions, this design only involved a single survey. Athletes who responded may have been an atypical or biased sampling of all roller derby players. Since the study predominantly disseminated through social media, social networks active in league- or interest-oriented groups may fail to include the full diversity of players. Respondents also were not subject to independent verification of their medical information or injury narratives (i.e., we had no direct access to medical records). However, this is not so different from how concussions frequently are diagnosed in the clinic, which is often done on the basis of self-report of subjective symptoms, even when the results of commonly used neuroimaging methods are unremarkable and neurological examination is normal (61). Online data collection is becoming an increasingly common and validated source of health and behavioral data (44, 46, 47). The design of this study did not permit us to determine if respondents had high intra-rater consistency. A weakness of this approach is that there was no practical means of following up with respondents to better understand ambiguous responses, incomplete information, or to ask follow-up questions that arose during analysis (common activities when patients are seen in person). One question that we failed to ask was whether athletes who reported a recent concussion had returned to play or were otherwise considered recovered at the time of the survey. While this has more influence on the cognitive and linguistic dimensions of the survey, it could potentially have impacted the quality of all responses by individuals with recent injuries, as using screens during this time is often challenging and may lead to an increase in symptoms, such as headache and light-sensitivity (62, 63).

Roller derby is an exciting sport that holds a position of great importance among many players, well beyond the end of a bout. Discourse samples describing the sport frequently mentioned community, empowerment, and support, echoing the extensive literature on the sport in gender studies and ethnography. We were unable to examine gender diversity and its impact on the sport of roller derby given the small number of participants but appreciate that this is an important direction for future work. Increased attention in recent years has facilitated a richer foundation for players to make informed decisions about participation; however, these findings raise as many questions as are answered, particularly regarding the impact of gender on cognitive sequelae of concussion, departure from the sport, and long-term consequences of injury. It is our hope that medical research will continue to examine this unique sport.

## Data Availability Statement

The original contributions presented in the study are publicly available. This data can be found here: Stockbridge M. English Stockbridge Narrative & Roller-Derby elicited written corpora. TBIBank2021. doi: 10.21415/D70T-C409.

## Ethics Statement

The studies involving human participants were reviewed and approved by University of Maryland Institutional Review Board. The patients/participants provided their written informed consent to participate in this study.

## Author Contributions

MS and RN conceived of the study and design. MS acquired the data, organized the data for analysis, and wrote the first draft of the manuscript. ZK and MS arrived at a statistical plan and performed the statistical analysis. All authors contributed to the manuscript revision and read and approved the submitted version.

## Funding

MS was supported by NIDCD P50 DC014664.

## Conflict of Interest

The authors declare that the research was conducted in the absence of any commercial or financial relationships that could be construed as a potential conflict of interest.

## Publisher's Note

All claims expressed in this article are solely those of the authors and do not necessarily represent those of their affiliated organizations, or those of the publisher, the editors and the reviewers. Any product that may be evaluated in this article, or claim that may be made by its manufacturer, is not guaranteed or endorsed by the publisher.

## Footnotes

^1^The Women's Flat Track Derby Association adopts a gender-expansive view of inclusion in the sport and permits any individual to skate with an associated team “if women's flat track roller derby is the version and composition of roller derby with which they most closely identify.” Players were recruited using materials and survey questions that specifically asked about *women's* roller derby participation. Given this inclusion criterion, we have not excluded players who identified as men on the survey from analysis, nor have we excluded any individual on the basis of reported identity. When describing other authors' findings, we have repeated the terms used by the authors themselves, as we cannot be certain which meaning they intended to convey.
